# Anisotropic Hyperelastic Strain Energy Function for Carbon Fiber Woven Fabrics

**DOI:** 10.3390/ma17102456

**Published:** 2024-05-20

**Authors:** Renye Cai, Heng Zhang, Chenxiang Lai, Zexin Yu, Xiangkun Zeng, Min Wu, Yankun Wang, Qisen Huang, Yiwei Zhu, Chunyu Kong

**Affiliations:** 1 School of Automobile and Transportation Engineering, Guangdong Polytechnic Normal University, Guangzhou 510665, China; cairenye@gpnu.edu.cn (R.C.); zhangh08200@126.com (H.Z.); wumin35045@126.com (M.W.); wyk@jlu.edu.cn (Y.W.); huangqs12138@126.com (Q.H.); ivykcy@hotmail.com (C.K.); 2 Guangdong Bangda Industrial Co., Ltd., Zhongshan 528400, China; 3 Shien-Ming Wu School of Intelligent Engineering, South China University of Technology, Guangzhou 510641, China; 4 Guangzhou Metro Design & Research Co., Ltd., Guangzhou 510010, China; laichenxiang@gmdi.cn; 5 Institute for Manufacturing Technologies of Ceramic Components and Composites (IMTCCC), University of Stuttgart, Allmandring 7b, 70569 Stuttgart, Germany; yu.zexin@ifkb.uni-stuttgart.de

**Keywords:** strain energy function (sef), anisotropic hyperelastic materials, large deformation, nonlinear mechanics, carbon fiber woven fabrics

## Abstract

The present paper introduces an innovative strain energy function (SEF) for incompressible anisotropic fiber-reinforced materials. This SEF is specifically designed to understand the mechanical behavior of carbon fiber-woven fabric. The considered model combines polyconvex invariants forming an integrity basisin polynomial form, which is inspired by the application of Noether’s theorem. A single solution can be obtained during the identification because of the relationship between the SEF we have constructed and the material parameters, which are linearly dependent. The six material parameters were precisely determined through a comparison between the closed-form solutions from our model and the corresponding tensile experimental data with different stretching ratios, with determination coefficients consistently reaching a remarkable value of 0.99. When considering only uniaxial tensile tests, our model can be simplified from a quadratic polynomial to a linear polynomial, thereby reducing the number of material parameters required from six to four, while the fidelity of the model’s predictive accuracy remains unaltered. The comparison between the results of numerical calculations and experiments proves the efficiency and accuracy of the method.

## 1. Introduction

Carbon fiber woven reinforcement materials have been widely used in many fields of automotive, aerospace, national defense, and civil industry, especially favored by new energy vehicles, owing to their superior mechanical characteristics, including high strength, high modulus, good quality, strong designability, and processability [1]. Carbon-woven fabric composites usually use resin as the matrix and carbon fiber as the reinforcement, which is generally considered to be a hyperelastic anisotropic material. It is also noted that the viscosity of resin and hardener mixtures, which remain liquid at room temperature and for a short time at high temperatures, has little impact on the forming mechanical properties of carbon fiber-woven fabrics. Fiber-reinforced woven fabrics, acting as the reinforcement in composite materials, not only serve to bear loads but also endow the composite with excellent formability and designability. Therefore, studying the mechanical behavior of woven fabrics during the forming process is conducive to advancing the development of stamping for woven composites. Currently, the mechanical properties and deformation mechanisms of fiber-reinforced composite materials are mainly studied through experimental measurements and theoretical modeling. Many material performance testing experiments were designed, commonly including uniaxial tensile tests, biaxial tensile tests, bending tests, and frame shear tests [2,3,4,5,6]. Cao et al. established a standard specification for mechanical performance experiments on composite materials [7].

In terms of theoretical modeling, the mechanical behavior of carbon fiber composites is mainly studied from the micro model, meso model, and macro model. On the microscopic level, a “kinematic model” has been established to analyze the stretchable properties of fiber-reinforced woven composites. This model is developed based on the combination of a unit cell model with microstructural parameters and the development of micromechanics, but it has high computational costs and cannot be applied to stamping analysis [8]. At the mesoscale, a single cell model for woven materials can effectively characterize the internal structure of composite materials or the mechanical behavior of a single fiber, but it cannot effectively indicate the performance of composite materials woven from a large number of fibers and is not suitable for analyzing their forming process [9]. At the macro level, phenomenological energy functions can be used to describe the macroscopic mechanics of fiber-reinforced hyperelastic materials, which can be mainly divided into statistical mechanical models, strain tensor component forms, and strain invariant forms. A common way to build a hyperelastic strain energy function is by using strain invariants. It has been shown that the strain energy function’s form should not be limited by any preconceived constraints [10,11]. That led to the expansion of the expression form of the SEF from early simple polynomials to different forms such as logarithmic, exponential, or power forms [12,13,14,15]. Aimene et al. have decomposed the strain energy of carbon fiber woven materials into two types of tensile energy and shear energy, successfully simulating the hemispherical stamping and forming of materials [16]. This mechanical model proposed by Islam et al. can predict the stress-strain behavior, deformation profile, and shear strain angle of fiber-reinforced composite materials when subjected to uniaxial tension [17]. Huang et al. [18] developed an SEF for woven composite reinforcements. This SEF is additively decomposed into components that represent the tensile energy resulting from fiber elongation, the compaction energy arising from the biaxial tension coupling in the warp and weft directions, and the shearing energy that stems from interactions between the fibers.

The models previously discussed, in common with the majority of studies in the academic literature, are often assumed to satisfy convexity/multi-convexity conditions to ensure the existence and unique solution of hyperelastic problems. However, in reality, many existing strain energy functions do not satisfy convexity conditions, which may lead to some numerical problems [19]. More recently, an original integrity basis made of polyconvex invariants was introduced by Cai et al. [20], which can be used to build SEF for anisotropic materials with two fiber families. This polyconvex integrity basis, inspired by the work of Ta et al. [21], is mathematically substantiated by the theory of invariant polynomials. One advantage of this new set of polyconvex invariants is that the SEF constructed with them is polyconvex, which is deemed essential to fulfilling the prerequisite that guarantees the existence of solutions aligned with the physical requirements [22]. It provides an alternative to the method of constructing the strain energy function SEF based on the classical invariants $I_{i}$ as found in the literature. In addition, traditional strain energy functions, which are composed of classical invariants, have some material parameters that lack physical meaning and are even more difficult to identify. These may lead to the inability of this constitutive model to be used for subsequent finite element implementation, limiting its application scope. The physical interpretation of these polyconvex invariants has been demonstrated in our previous work [20]. However, to the best of our knowledge, the invariants introduced by Cai et al. [20] have not yet been widely applied in practice. Currently, the application of this set of invariants is primarily seen in the simulation of orthotropic biological soft tissues, such as the responses of the peripheral arteries and the passive ventricular myocardium [20,23]. Given the incompressibility of the considered materials, how could the proposed SEF be incorporated into a finite element code? This is a key issue for the widespread application of this SEF. Kakavas et al. present a mixed finite element formulation for approximating the large deformations observed in the analysis of elastomeric butt-joints [24]. In our previous work, considering the incompressibility of the material, we introduced a penalty function, and it has been successfully demonstrated that the finite element implementation of the polynomial strain energy function (SEF) constructed with this set of polyconvex invariants has been successfully executed within the FER University code [25]. So, the main purpose of this article is to broaden the application scope of these polyconvex invariants to model the behaviors of carbon fiber-woven materials. In Section 2, we conduct uniaxial tensile tests on carbon fiber-woven materials to explore their nonlinear and anisotropic mechanical properties under large deformation conditions, providing a foundation for material parameters necessary for the subsequent development of a constitutive model. To validate the precision and applicability of our models, we have compared the predicted outcomes with experimental data obtained from these uniaxial tensile and biaxial tensile experiments obtained from the work of Huang et al. [26] with different stretching strain ratios ($k=\frac{\varepsilon_{weft}}{\varepsilon_{warp}}$, *k* = 2, 1, 0.5) applied to the warp and weft directions, respectively. We proposed a new strain energy function based on the polyconvex invariants introduced in Cai et al. [20] in quadratic polynomial form. Our model, which includes six material parameters, is capable of predicting biaxial tensile experimental data with different stretching strain ratios. Distinguishing from traditional anisotropic models that employ a case-sensitive material parameter to characterize the tension and compression states of fibers, which may introduce discontinuities in the stress tensor, our model ensures the continuity of stress. Considering specific tensile scenarios, the number of material parameters can be further reduced. For example, in Section 4.1, we demonstrate that under uniaxial tensile loading, our model can be simplified from six to four material parameters, with the model taking on a linear polynomial form.

### Notations

A bold lowercase Latin letter, for instance  a, represents a vector, while a bold uppercase Latin letter, such as ***A***, signifies a second-order tensor. The standard Euclidean scalar product is denoted by a pair of double vertical bars:

$$\langle Aa,a\rangle =\sum_{i=1}^{3}A_{ij}a_{ja_{i}}$$

The tensor product of two vectors, a and b, is defined as follows:

$${\left(a⨂b\right)}_{ij}=a_{i}b_{j}$$

## 2. Materials and Methods

### 2.1. Sample and Test

**Figure 1:**
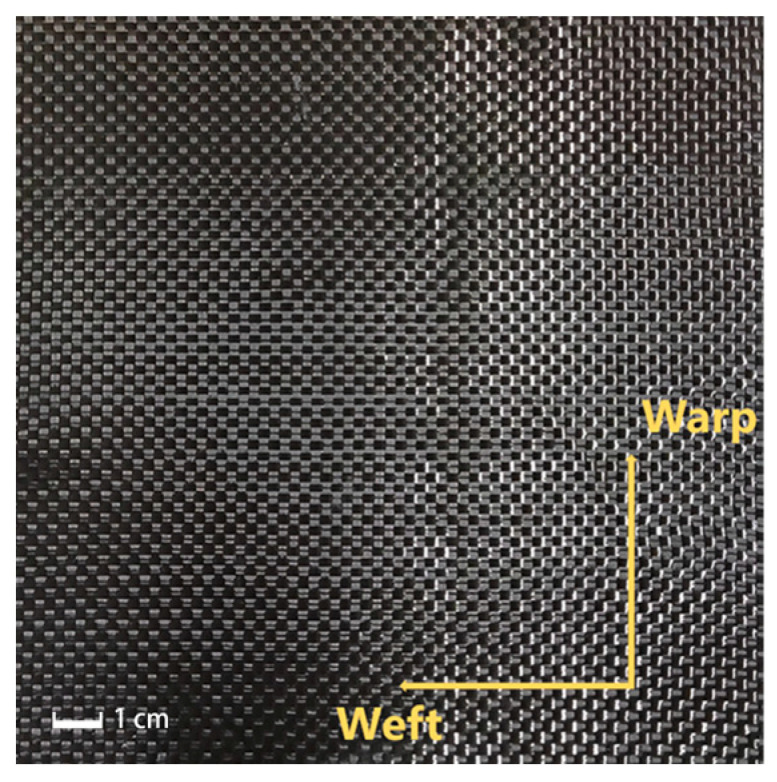
2D braided fabric structure.

Considering the nonlinear and anisotropic mechanical properties that woven fabrics exhibit during the forming process, in this paper, we perform uniaxial tensile tests on carbon fiber woven materials to explore their mechanical behavior and deformation mechanisms under conditions of large deformation. We focus on the plain weave carbon fiber fabric, where the fiber strands are arranged in a simple alternating up/down pattern. The directions of the warp and weft yarns are denoted as $d_{warp}$ and $d_{weft}$, respectively, as shown in Figure 1.

**Figure 2:**
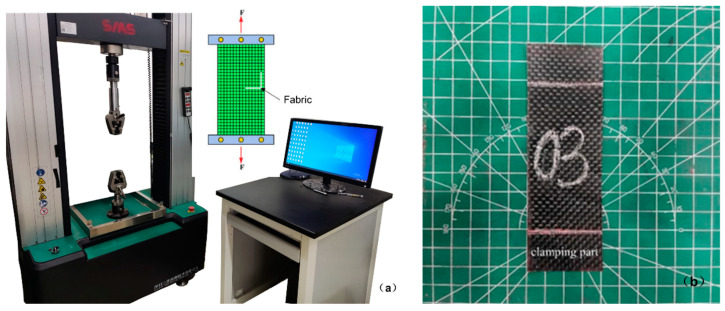
Uniaxial tensile and test specimen. (**a**) Uniaxial stretching equipment, (**b**) test specimen.

To assess the tensile mechanical properties of plain-woven carbon fiber materials, a uniaxial tensile test was performed using an EUT5000 universal testing machine (Shenzhen Sansi Testing Co., Ltd., Shenzhen, China), as shown in Figure 2a. The 12 K carbon fiber fabric (CF) utilized in this study was obtained from Toray Industries, Japan, with a weight of 200 $g/m^{2}$ (referenced as material A). It was noted that the gripping range of the clamp is 20 × 40 mm. To match the width of the equipment jaws, the width of the specimen is set to 40 mm. Uniaxial tensile tests were performed on a carbon fiber woven fabric sample. The effective size of the sample is 80×40 mm, the length of each gripping section is 20 mm. For the uniaxial testing, the thickness of each sample was determined by measuring at six different random sites, with an average thickness recorded as 0.5 mm. The test axes were aligned with the warp direction of the sample. The sample was secured to the testing apparatus using clamps, with sandpaper affixed to each end to prevent slipping, as shown in Figure 2b.

**Figure 3:**
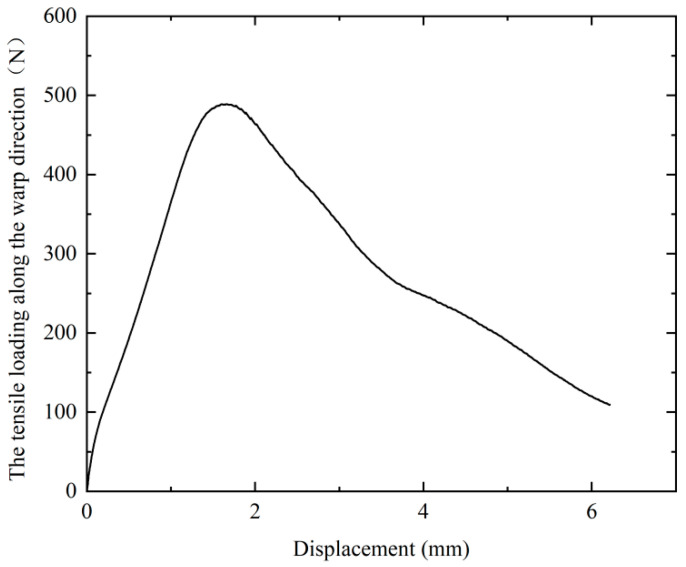
Load-displacement curve obtained from the test.

The experiment was conducted at 20 °C with a stretching speed of 2 mm/min. Six sets of tensile tests were conducted, and the resulting force-displacement data were processed by averaging, with the final results depicted as shown in Figure 3. At first, as the displacement increases, the applied force also increases. However, when displacement increases to 1.8 mm, the force sharply decreases because fiber fracture occurs during this stage. The tensile properties of carbon fiber-woven fabrics along the fiber direction exhibit highly nonlinear mechanical behavior. Therefore, when identifying material parameters, we only use experimental data before fiber fracture to compare with our model.

**Figure 4:**
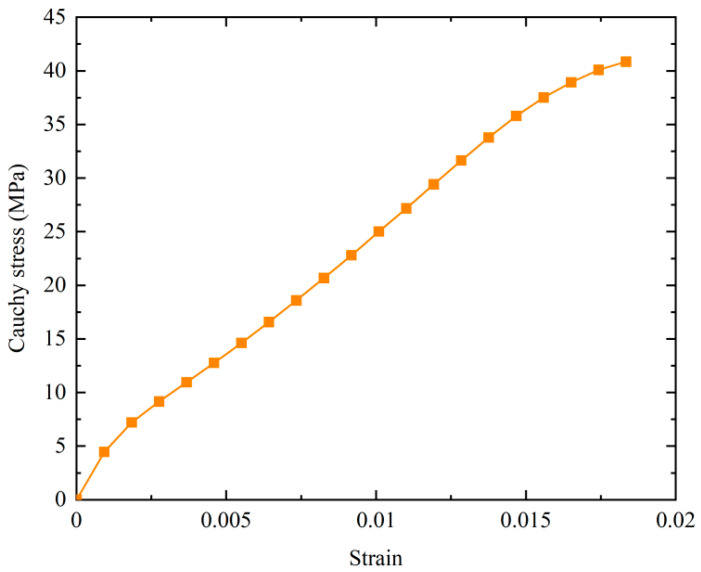
Cauchy stress-strain curve in the uni-axial tensile test.

The Cauchy stresses were calculated as a function of the applied stretch in the warp direction of sample as:

(1) $$\sigma_{warp}^{exp}=\frac{F}{h_{exp}l_{weft}\;\;}$$

where $h_{exp}$ is the deformed thickness of the sample in the current configuration during the uniaxial test, F is applied forces in the warp direction, and $l_{weft}$ is the deformed lengths over which these forces act. Specifically, the local stretch ratio ($\lambda_{warp}$) was calculated based on $\lambda_{warp}=\frac{l_{warp}}{L_{warp}}$, while $l_{warp}$ is the deformed lengths over which these forces act and $L_{warp}$ is the distance in the warp direction at rest. Based on the assumption of incompressibility, the Cauchy stress $\sigma_{warp}^{exp}$ was calculated as

(2) $$\sigma_{warp}^{exp}==\frac{F\lambda_{warp}}{H_{exp}L_{weft}}$$

where $H_{exp}$ is the thickness of the sample at rest, and $L_{weft}$ is the distance in the weft direction at rest. Therefore, the stress and strain of the sample can be calculated, and the constitutive relationship of the material is shown in Figure 4. It can also be seen that the material exhibits nonlinear properties.

### 2.2. Preliminaries

**Figure 5:**
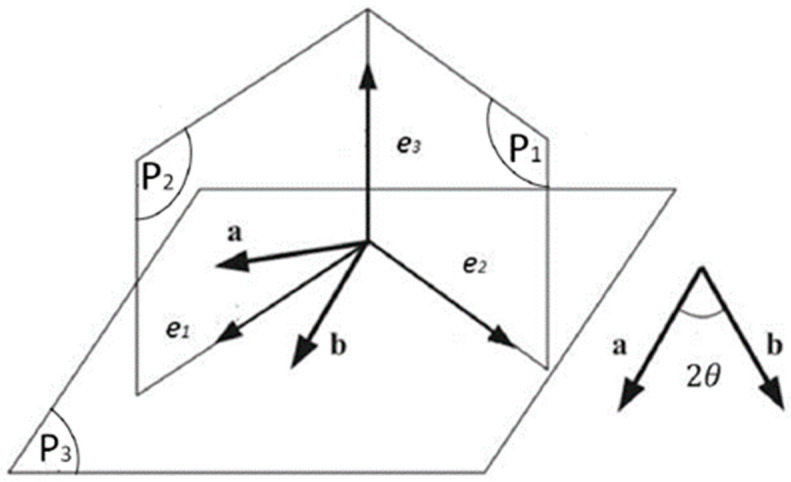
The material plane of symmetry.

In the work of Ta et al. [21], a material symmetry group $S_{8}$ has been identified for fiber-reinforced materials with two fiber families. It is assumed that the two fiber directions, denoted as ***a*** and ***b***, are situated within the plane $P_{3}$ defined by vectors $e_{1}$ and $e_{2}$. These two fiber directions are symmetrically distributed along vector $e_{1}$, as illustrated in Figure 5. Vectors $e_{1}$, $e_{2}$, and $e_{3}$ form an orthogonal coordinate system; plane $P_{2}$ contains the co-bisector $e_{2}$ of a and b; and plane $P_{3}\;$is generated by $e_{1}$ and $e_{3}$.

This group is composed of 8 invariant matrix operators, including the three reflections related to planes $P_{1}$, $P_{2}$ and $P_{3}$, three rotational operators performing a π radians rotation around the axes $e_{1}$, $e_{2}$ and $e_{3}$, as well as the identity matrix I and its negation −I. They have shown that seven polynomial invariants constitute a complete set within the ring of invariant polynomials for the material symmetry group $S_{8}$.

(3) $$\begin{array}{c} K_{1}=\rho_{1};\;\;K_{2}=\rho_{2};\;\;K_{3}=\rho_{3};\;\;K_{4}=\rho_{4}^{2};\\ K_{5}=\rho_{5}^{2};\;\;K_{6}=\rho_{6}^{2};\;\;K_{7}=\rho_{4}\rho_{5}\rho_{6} \end{array}$$

where the coefficients $\rho_{i}$ stand for

(4) $$\begin{array}{c} \rho_{1}=\langle {Ce}_{1},e_{1}\rangle ;\;\;\;\rho_{2}=\langle {Ce}_{2},e_{2}\rangle ;\;\;\;\rho_{3}=\langle {Ce}_{3},e_{3}\rangle \\ \rho_{4}=\langle {Ce}_{1},e_{2}\rangle ;\;\;\;\rho_{5}=\langle {Ce}_{1},e_{3}\rangle ;\;\;\;\rho_{6}=\langle {Ce}_{2},e_{3}\rangle \end{array}$$

The tensor C is recognized as the classical Right Cauchy–Green deformation tensor.

(5) $$C=F^{T}F$$

The transformation gradient tensor, denoted as F, is defined by the following relationship:

(6) $$F=\frac{\partial x}{\partial X}=I+\frac{\partial u}{\partial X}$$

where x and X denote the current and reference positions, respectively, of a material point, while u represents the displacement vector.

However, not every one of these invariants is polyconvex. To address this issue, Cai et al. [20] proposed a set of 7 polyconvex invariants that constitute an integrity basis:

(7) $$\begin{array}{c} L_{1}=Tr\left(Ce_{1}⨂e_{1}\right);\;\;\;\;\;\;L_{2}=Tr\left(Ce_{2}⨂e_{2}\right);\;\;\;\;\;L_{3}=Tr\left(Ce_{3}⨂e_{3}\right)\\ L_{4}={\left(L_{1}+L_{2}\right)}^{2}+4{\left\langle Ce_{1},e_{2}\right\rangle }^{2};\;\\ L_{5}={\left(L_{1}+L_{3}\right)}^{2}+4{\left\langle Ce_{1},e_{3}\right\rangle }^{2}\\ L_{6}={\left(L_{2}+L_{3}\right)}^{2}+4{\left\langle Ce_{2},e_{3}\right\rangle }^{2}\\ L_{7}=\left\langle Ce_{1},e_{2}\right\rangle \left\langle Ce_{1},e_{3}\right\rangle \left\langle Ce_{2},e_{3}\right\rangle +\frac{1}{2}\left\{L_{1}L_{2}L_{3}-L_{1}{\left\langle Ce_{2},e_{3}\right\rangle }^{2}-L_{2}{\left\langle Ce_{1},e_{3}\right\rangle }^{2}-L_{3}{\left\langle Ce_{1},e_{2}\right\rangle }^{2}\right\}\;\; \end{array}$$

It should be noted that plain weave carbon fiber woven fabric also satisfies the condition of material symmetry group $S_{8}$ proposed by Ta et al. [21], if the directions $d_{warp}$ and $d_{weft}$ follow $e_{1}$ and $e_{2}$ respectively. Therefore, the invariants defined by Equation (7) can be used to define our SEF for plain weave carbon fiber woven fabric. Moreover, it was also proved in our previous work that $L_{7}$ is correlated with the additional pressure p, which is a redundant term in the formula [20]. So only the initial six polyconvex invariants, namely $L_{1}$ through $L_{6}$, should be taken into account when constructing the strain energy function *W*.

(8) $$W=W(L_{1},L_{2},L_{3},L_{4},L_{5},L_{6})$$

Regarding the stress tensors, the second Piola–Kirchhoff stress tensor, denoted as S, and its corresponding Cauchy stress tensor, denoted as σ, can be articulated as follows:

(9) $$S=\frac{\partial W}{\partial E}=2\frac{\partial W}{\partial C}-pC^{-1}$$

(10) $$\sigma =J^{-1}FSF^{T}$$

An additional pressure term p is integrated into the formulation to fulfill the incompressibility condition $J=\det\left(F\right)=1$. Substituting Equation (9) into Equation (10) yields the following:

(11) $$\sigma ={2J}^{-1}F\frac{\partial W}{\partial C}F^{T}-pI={2J}^{-1}F\omega_{i}\frac{\partial L_{i}}{\partial C}F^{T}-pI$$

The derivatives $\frac{{\partial L}_{i}}{\partial C}$, which are embedded in Equation (11), can be calculated based on Equation (7):

(12) $$\begin{array}{c} \frac{{\partial L}_{1}}{\partial C}=e_{1}⨂e_{1}\;;\;\;\;\frac{{\partial L}_{2}}{\partial C}=e_{2}⨂e_{2}\;;\;\;\;\frac{{\partial L}_{3}}{\partial C}=e_{3}⨂e_{3}\\ \frac{{\partial L}_{4}}{\partial C}=2\left\{\left(L_{1}+L_{2}\right)\left(e_{1}⨂e_{1}+e_{2}⨂e_{2}\right)+\sqrt{L_{4}-{\left(L_{1}+L_{2}\right)}^{2}}(e_{1}⨂e_{2}+e_{2}⨂e_{1})\right\}\\ \frac{{\partial L}_{5}}{\partial C}=2\left\{\left(L_{1}+L_{3}\right)\left(e_{1}⨂e_{1}+e_{3}⨂e_{3}\right)+\sqrt{L_{5}-{\left(L_{1}+L_{3}\right)}^{2}}(e_{1}⨂e_{3}+e_{3}⨂e_{1})\right\}\\ \frac{{\partial L}_{6}}{\partial C}=2\left\{\left(L_{2}+L_{3}\right)\left(e_{2}⨂e_{2}+e_{3}⨂e_{3}\right)+\sqrt{L_{6}-{\left(L_{2}+L_{3}\right)}^{2}}(e_{2}⨂e_{3}+e_{3}⨂e_{2})\right\} \end{array}$$

To derive the partial derivatives $\omega_{i}=\frac{\partial W}{\partial L_{i}}$, featured in Equation (11), it is crucial to establish a method for constructing a suitable strain energy function W based on these invariants $L_{i}$. This will be discussed in the subsequent sections.

## 3. Homogeneous Deformations

**Figure 6:**
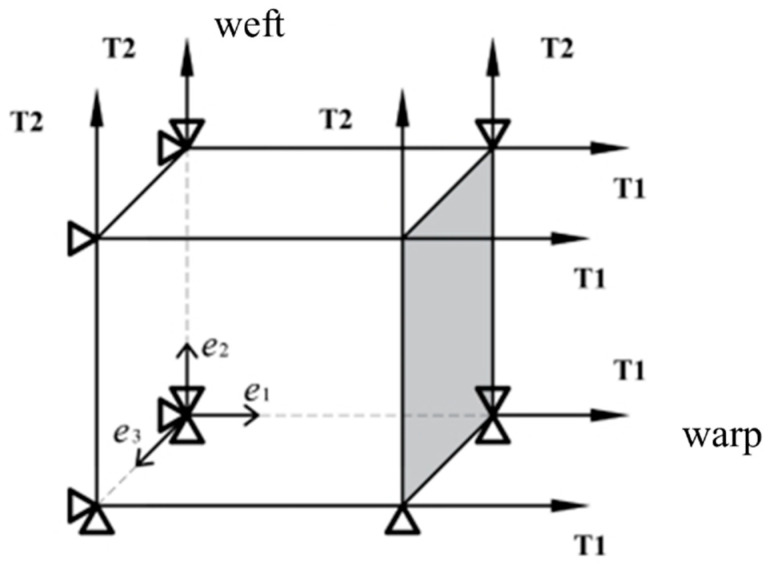
The boundary conditions for the biaxial tension test.

Since the experimental data discussed in this paper is related to uniaxial and biaxial tensile tests, we will derive the constitutive relationship during tensile loading in this section. Consider biaxial stretching, as depicted in Figure 6, where loading is applied to both the warp and weft directions with distinct ratios. The ratio is 0 in the case of uniaxial tensile. These boundary conditions result in the subsequent homogenous deformation:

(13) $$F=\left(\begin{array}{ccc} \lambda_{1} & 0 & 0\\ 0 & \lambda_{2} & 0\\ 0 & 0 & \lambda_{3} \end{array}\right)\Rightarrow C=\left(\begin{array}{ccc} \lambda_{1}^{2} & 0 & 0\\ 0 & \lambda_{2}^{2} & 0\\ 0 & 0 & \lambda_{3}^{2} \end{array}\right)$$

where $\lambda_{1}$, $\lambda_{2},$ and $\lambda_{3}$ represent the principal stretches. The incompressibility condition is satisfied by $J=\det\left(F\right)=\lambda_{1}\lambda_{2}\lambda_{3}=1$.

Substituting the components of C from Equation (13) into Equation (7) yields the expressions for the six polyconvex invariants:

(14) $$\begin{array}{c} L_{1}=\lambda_{1}^{2}\;;\;\;L_{2}=\lambda_{2}^{2};\;L_{3}=\lambda_{1}^{-2}\lambda_{2}^{-2}\\ \;L_{4}={\left(\lambda_{1}^{2}+\lambda_{2}^{2}\right)}^{2}\;;\;L_{5}={\left(\lambda_{1}^{2}+\lambda_{1}^{-2}\lambda_{2}^{-2}\right)}^{2}\;;\;L_{6}={\left(\lambda_{2}^{2}+\lambda_{1}^{-2}\lambda_{2}^{-2}\right)}^{2} \end{array}$$

The derivatives $\frac{{\partial L}_{i}}{\partial C}$ can be expressed as:

(15) $$\begin{array}{c} \frac{{\partial L}_{4}}{\partial C}=2(\lambda_{1}^{2}+\lambda_{2}^{2})(e_{1}⨂e_{1}+e_{2}⨂e_{2})\\ \frac{{\partial L}_{5}}{\partial C}=2(\lambda_{1}^{2}+\lambda_{1}^{-2}\lambda_{2}^{-2})(e_{1}⨂e_{1}+e_{3}⨂e_{3})\\ \frac{{\partial L}_{6}}{\partial C}=2(\lambda_{2}^{2}+\lambda_{1}^{-2}\lambda_{2}^{-2})(e_{2}⨂e_{2}+e_{3}⨂e_{3}) \end{array}$$

We next report Equations (12), (13) and (15) in Equation (11) to derive the three diagonal components of the Cauchy stress tensor, with all other components being zero:

(16) $$\sigma_{11}=2\lambda_{1}^{2}\left\{w_{1}+2w_{4}\left(\lambda_{1}^{2}+\lambda_{2}^{2}\right)+2w_{5}\left(\lambda_{1}^{2}+\lambda_{1}^{-2}\lambda_{2}^{-2}\right)\right\}-p$$

(17) $$\sigma_{22}=2\lambda_{2}^{2}\left\{w_{2}+2w_{4}\left(\lambda_{1}^{2}+\lambda_{2}^{2}\right)+2w_{6}\left(\lambda_{2}^{2}+\lambda_{1}^{-2}\lambda_{2}^{-2}\right)\right\}-p$$

(18) $$\sigma_{33}=2\lambda_{1}^{-2}\lambda_{2}^{-2}\left\{w_{3}+2w_{5}\left(\lambda_{1}^{2}+\lambda_{1}^{-2}\lambda_{2}^{-2}\right)+2w_{6}\left(\lambda_{2}^{2}+\lambda_{1}^{-2}\lambda_{2}^{-2}\right)\right\}-p$$

The free loading condition $\sigma_{33}=0$ is finally used with Equation (18) for eliminating the extra pressure p from Equations (16) and (17):

(19) $$\sigma_{11}=2\left\{w_{1}\lambda_{1}^{2}-w_{3}\lambda_{1}^{-2}\lambda_{2}^{-2}+2w_{4}\left(\lambda_{1}^{4}+{\lambda_{1}^{2}\lambda }_{2}^{2}\right)+2w_{5}\left(\lambda_{1}^{4}-\lambda_{1}^{-4}\lambda_{2}^{-4}\right)-2w_{6}\left(\lambda_{1}^{-2}+\lambda_{1}^{-4}\lambda_{2}^{-4}\right)\right\}$$

(20) $$\sigma_{22}=2\left\{w_{2}\lambda_{2}^{2}-w_{3}\lambda_{1}^{-2}\lambda_{2}^{-2}+2w_{4}\left(\lambda_{2}^{4}+\lambda_{1}^{2}\lambda_{2}^{2}\right)-2w_{5}\left(\lambda_{2}^{-2}+\lambda_{1}^{-4}\lambda_{2}^{-4}\right)+2w_{6}\left(\lambda_{2}^{4}-\lambda_{1}^{-4}\lambda_{2}^{-4}\right)\right\}$$

Equations (19) and (20) furnish a closed-form solution for the homogeneous tension test illustrated in Figure 6. The material parameters will be obtained by comparing these closed-form solutions with their corresponding experimental values. When considering the situation of uniaxial tension along the warp direction, which means $\;T_{2}=0$, it is obvious that $\sigma_{22}=0$.

## 4. Results and Discussion

### 4.1. A New Hyperelastic Model

Adhering to the approach established by Mooney and Rivlin for constructing isotropic energy densities [27,28], this study employs a polynomial expression for the strain energy function W. A significant advantage of utilizing this polynomial form is the considerable simplification it offers in the identification of the model’s material parameters. To evaluate if our model could be appropriate, a tensile test and biaxial tensile test of experimental data of different plain weave carbon fiber-woven fabrics were compared with our closed-form solution.

#### Linear Strain Energy Density

We propose a linear polynomial formulation with respect to $L_{1},L_{3},L_{4},L_{5},\;$and $L_{6}$:

(21) $$W_{1}=a_{1}L_{1}+a_{2}L_{2}{+a}_{3}L_{3}+a_{4}L_{4}+a_{5}L_{5}+a_{6}L_{6}$$

The six polynomial coefficients $a_{1}$, $a_{2}$, $a_{3}$, $a_{4}$, $a_{5}$, and $a_{6}$ represent the material parameters. The derivatives $w_{i}=\frac{\partial W}{\partial L_{i}}$ can be directly calculated:

(22) $$\frac{\partial W}{\partial L_{1}}=a_{1};\;\;\frac{\partial W}{\partial L_{2}}=a_{2};\;\frac{\partial W}{\partial L_{3}}=a_{3};\;\frac{\partial W}{\partial L_{4}}=a_{4};\;\frac{\partial W}{\partial L_{5}}=a_{5};\;\frac{\partial W}{\partial L_{6}}=a_{6}$$

In the reference configuration, the displacement field is null, means F=C=I, the Equations (4), (7), (11), (12) and (15) can be expressed as:

(23) $$\rho_{1}=\rho_{2}=\rho_{3}=1,\;\rho_{4}=\rho_{5}=\rho_{6}=0$$

(24) $$L_{1}=L_{2}=L_{3}=1,\;\rho_{4}=\rho_{5}=\rho_{6}=0$$

(25) $$\frac{{\partial L}_{1}}{\partial C}=e_{1}⨂e_{1}\;\;\;\;;\;\;\;\frac{{\partial L}_{3}}{\partial C}=e_{3}⨂e_{3}$$

(26) $$\frac{{\partial L}_{4}}{\partial C}=4(e_{1}⨂e_{1}+e_{2}⨂e_{2}),\;\frac{{\partial L}_{5}}{\partial C}=4(e_{1}⨂e_{1}+e_{3}⨂e_{3}),\frac{{\partial L}_{6}}{\partial C}=4(e_{2}⨂e_{2}+e_{3}⨂e_{3})$$

(27) $$\sigma =2\left(\begin{array}{ccc} a_{1}+4a_{4}+4a_{5} & 0 & 0\\ 0 & a_{2}+4a_{4}+4a_{6} & 0\\ 0 & 0 & a_{3}+4a_{5}+4a_{6} \end{array}\right)-pI$$

Finally, by accounting the fact that σ=0 in the reference configuration, it is possible to express $a_{1}$ and $a_{2}$ in terms of the remaining material parameters:

(28) $$a_{1}=a_{3}-4a_{4}+4a_{6}a_{2}=a_{3}-4a_{4}+4a_{5}$$

Reporting Equation (28) in Equations (19) and (20) yields to the final expression of the Cauchy stress components $\sigma_{11}$ and $\sigma_{22}$, which depend only on five material parameters:

(29) $$\sigma_{11}=2a_{3}\left(\lambda_{1}^{2}-\lambda_{1}^{-2}\lambda_{2}^{-2}\right)+4a_{4}\left(\lambda_{1}^{4}+{\lambda_{1}^{2}\lambda }_{2}^{2}-2\lambda_{1}^{2}\right)+4a_{5}\left(\lambda_{1}^{4}-\lambda_{1}^{-4}\lambda_{2}^{-4}\right)+4a_{6}\left(2\lambda_{1}^{2}-\lambda_{1}^{-2}-\lambda_{1}^{-4}\lambda_{2}^{-4}\right)$$

(30) $$\sigma_{22}=2a_{3}\left(\lambda_{2}^{2}-\lambda_{1}^{-2}\lambda_{2}^{-2}\right)+4a_{4}\left(\lambda_{2}^{4}+{\lambda_{1}^{2}\lambda }_{2}^{2}-2\lambda_{2}^{2}\right)+4a_{5}\left(2\lambda_{2}^{2}-\lambda_{2}^{-2}-\lambda_{1}^{-4}\lambda_{2}^{-4}\right)+4a_{6}\left(\lambda_{2}^{4}-\lambda_{1}^{-4}\lambda_{2}^{-4}\right)$$

In the case of uniaxial tensile tests along the warp direction of carbon fiber-woven fabric, that means no force is applied in the weft direction ($T_{2}=0$). Thus, the free boundary condition $\sigma_{22}=0$ transforms Equation (30) into a fourth-degree polynomial equation this time. Utilizing the fzero function from the MATLAB software (https://www.mathworks.com/products/matlab.html), we have solved this equation and substituted the numerical solution $\lambda_{2}$ into Equation (29).

Was selected to obtain the best agreement between the theoretical results and the measurements. To identify this set, we have used the classical coefficient of determination $R^{2}$:

(31) $$R^{2}=1-\frac{S_{res}}{S_{tot}}$$

where $S_{res}$ and $S_{tot}$ are the residual sum of squares and the total sum of squares, respectively, calculated over the n experimental data points:

(32) $$S_{res}={\left\|y-f\right\|}^{2}=\sum_{i=1}^{n}{\left(y_{i}-f_{i}\right)}^{2};\;S_{tot}={\left\|y-\bar{y}\right\|}^{2}=\sum_{i=1}^{n}{\left(y_{i}-\bar{y}\right)}^{2}$$

The symbol $y_{i}$ represents the experimental data, $f_{i}$ denotes the theoretical data, and $\bar{y}$ signifies the mean of the experimental data:

(33) $$\bar{y}=\frac{1}{n}\sum_{i=1}^{n}y_{i}$$

**Figure 7:**
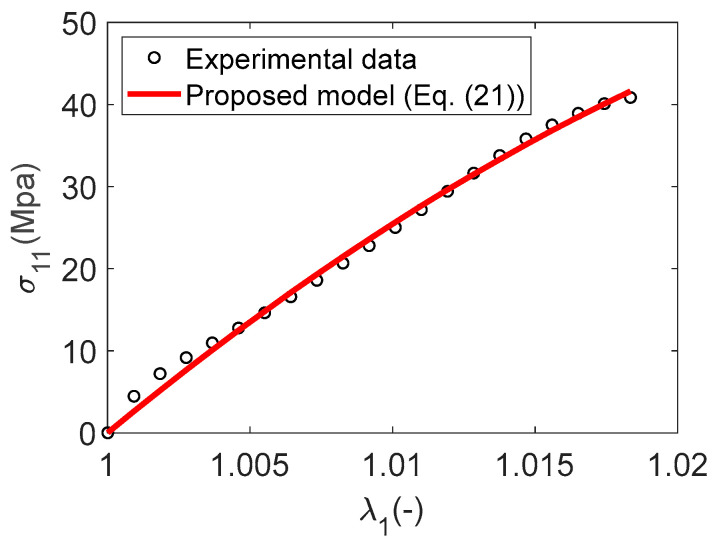
Material A: A comparison between the numerical result and the experimental data-linear case.

The closer the coefficient of determination $R^{2}$ is to 1, the better the fit of the theoretical data to the experimental data. Our objective, therefore, is to identify the set of material parameters that minimizes the ratio $\frac{S_{res}}{S_{tot}}$. The data fitting was executed using the same methods introduced by Cai et al. [20] for identifying material parameters on the Matlab platform. The identified parameters and the coefficient of determination $R^{2}$ for Material A are detailed in Table 1. The comparisons between the experimental and numerical results from Equation (21) are depicted in Figure 7. The results show better agreement between predicted and measured Cauchy stress curves. The equally good fitting effect can also be seen from the coefficient of determination $R^{2}=0.99$, which is close to 1.

To verify the applicability of this set of invariants in constructing strain energy functions for carbon fiber-woven fabrics, we compared our model results with different experimental tests. The experimental data, as reported by Huang et al. [26], pertain to samples that were examined using a specially fabricated biaxial testing apparatus. The sample was obtained by cutting carbon fiber T300-3K plain weave fabric (referenced as material B). Based on the geometric dimensions of the sample, the experimental Cauchy stress component can be calculated from the known tensile force and strain curves in their work [26]. The carbon fiber woven fabric sample underwent biaxial stretching tests with varying strain ratios, denoted as $k=\frac{\varepsilon_{weft}}{\varepsilon_{warp}}$, applied to the warp and weft directions. The strain ratios tested were in the proportions of 2:1, 1:1, and 1:2. The parameters corresponding to the strain energy density function in Equation (21) were subsequently identified, and the coefficients of determination $R^{2}$ in different stretching tests are presented in Table 2.

**Figure 8:**
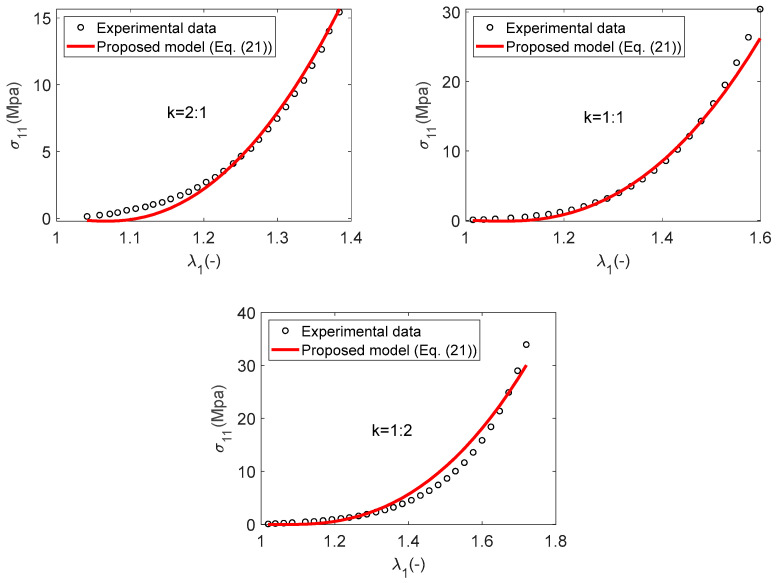
Material B: A comparison between the numerical result and the experimental data-linear case.

The comparison of experimental data with numerical predictions from the constitutive model, as defined by Equation (21), is depicted in Figure 8 for Material B. The results of model analysis were verified by the results of experiments, but there are still discrepancies in some areas, especially in the cases of strain-stretch ratios of k=1:2. This can also be observed from the value of the determination coefficient $R^{2}$, which is 0.97.

### 4.2. Quadratic Strain Energy Density

To enhance the predictive accuracy, especially for biaxial stretching scenarios, we have incorporated a quadratic polynomial expression for the strain energy density. It is noteworthy that $L_{1}$ and $L_{2}$ correspond to the square of the elongation in the $e_{1}$ and $e_{2}$ directions, respectively. When we consider biaxial tensile tests of materials along the warp and weft directions, respectively, the warp and weft mechanical properties of plain weave fabrics are equivalent [26]. To reduce the complexity of the model, only one invariant from *L*_1_ or *L*_2_ is needed to construct the strain energy function:

(34) $$W_{2}=a_{1}L_{1}{+a}_{3}L_{3}+a_{4}L_{4}+a_{5}L_{5}+a_{6}L_{6}+a_{7}L_{1}^{2}+a_{8}L_{3}^{2}$$

The derivatives $w_{i}=\frac{\partial W}{\partial L_{i}}$ can be directly calculated:

(35) $$\frac{\partial W}{\partial L_{1}}=a_{1}+2a_{7}L_{1};\;\frac{\partial W}{\partial L_{2}}=0;\;\frac{\partial W}{\partial L_{3}}=a_{3}+2a_{8}L_{3};\;\frac{\partial W}{\partial L_{4}}=a_{4};\;\frac{\partial W}{\partial L_{5}}=a_{5};\;\frac{\partial W}{\partial L_{6}}=a_{6}$$

We next report Equation (35) in the Equations (19) and (20), the Cauchy stress components $\sigma_{11}$ and $\sigma_{22}$ can written as:

(36) $$\sigma_{11}=2\left\{\left(a_{1}+2a_{7}\lambda_{1}^{2}\right)\lambda_{1}^{2}-\left(a_{3}+2a_{8}\lambda_{1}^{-2}\lambda_{2}^{-2}\right)\lambda_{1}^{-2}\lambda_{2}^{-2}+2a_{4}\left(\lambda_{1}^{4}+{\lambda_{1}^{2}\lambda }_{2}^{2}\right)+2a_{5}\left(\lambda_{1}^{4}-\lambda_{1}^{-4}\lambda_{2}^{-4}\right)-2a_{6}\left(\lambda_{1}^{-2}+\lambda_{1}^{-4}\lambda_{2}^{-4}\right)\right\}$$

(37) $$\sigma_{22}=2\left\{-\left(\left(a_{3}+2a_{8}\lambda_{1}^{-2}\lambda_{2}^{-2}\right)\right)\lambda_{1}^{-2}\lambda_{2}^{-2}+2a_{4}\left(\lambda_{2}^{4}+\lambda_{1}^{2}\lambda_{2}^{2}\right)-2a_{5}\left(\lambda_{2}^{-2}+\lambda_{1}^{-4}\lambda_{2}^{-4}\right)+2a_{6}\left(\lambda_{2}^{4}-\lambda_{1}^{-4}\lambda_{2}^{-4}\right)\right\}$$

Similar to the method we used in linear case, we can obtain the following relationship in the reference configuration:

(38) $$a_{1}=a_{3}-4a_{4}+4a_{6}-2a_{7}+2a_{8}$$

We concentrate on biaxial stretching with varying strain ratios applied to the warp and weft directions, specifically using the proportions of 2:1, 1:1, and 1:2. The six polynomial coefficients for the SEF, as outlined by Equation (34), have been determined using the same methodology as detailed in the preceding section, with the aim of optimizing the classical coefficient of determination $R^{2}$. These identified coefficients are subsequently presented in Table 3.

**Figure 9:**
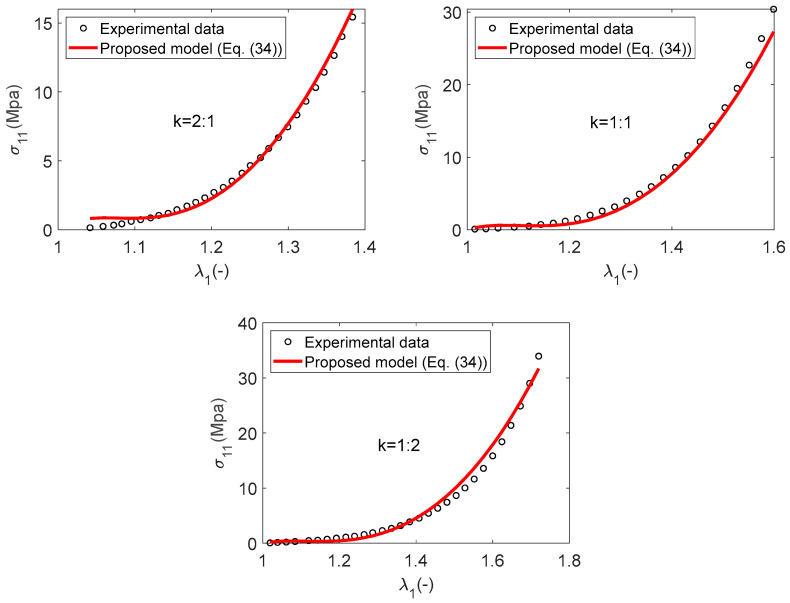
Material B: A comparison between the numerical result and the experimental data–quadratic case.

Figure 9 demonstrates a strong correlation between the numerical results and the experimental data, indicating a good agreement. It is observed that the quadratic model significantly enhances the precision of the numerical predictions, especially for the tensile loading scenario with a strain ratio of k=1:2. This enhancement is further substantiated by the increase in coefficient of determination $R^{2}$ from the SEF $W_{1}$ to the SEF $W_{2}$, where the value rises from 0.97 to 0.99. It is generally accepted that an $R^{2}$ value of 0.99 or higher denotes a highly satisfactory fit to the experimental data.

## 5. Conclusions

In this study, a novel anisotropic strain energy function (SEF) is developed for modeling plain weave carbon fiber woven fabric. This SEF is constructed upon a set of seven novel polyconvex invariants recently introduced by Cai et al. [20], utilizing a polynomial combination of these invariants. This ensures that the SEFs we construct have polyconvexity, a necessary condition for guaranteeing the existence of solutions that align with physical requirements. Our model diverges from traditional anisotropic models by not utilizing a case-sensitive material parameter that characterizes the tension and compression states of fibers. This particular setting guarantees the continuity of stress, which is advantageous for the subsequent finite element implementation within the FER code. One significant advantage of this method is its capability to perform least squares minimization, thereby yielding a unique set of material parameters. Because the SEF we constructed is a linear form of material parameters, we have confirmed that if we only consider the case of uniaxial tensile loading, our linear models defined by Equation (21) require only four material parameters to accurately capture the mechanical behavior of carbon fiber-woven fabric, with a determination coefficient of 0.99. However, for biaxial tensile deformation, discrepancies persist between the predictive outcomes and the experimental data, especially under conditions of equibiaxial and stretching strain ratios $k=\frac{\varepsilon_{weft}}{\varepsilon_{warp}}=0.5\;$ applied to the warp and weft directions. The determination coefficients $R^{2\;}$for the predictive model correspondingly decreased to 0.98 and 0.97, respectively. And we have demonstrated that the accuracy of predictions can be enhanced by elevating the polynomial’s degree, thereby transitioning from a linear model to a quadratic one defined by Equation (34). This quadratic polynomial function requires only six material parameters to accurately predict the mechanical response of carbon fiber-woven fabric, with the determination coefficients $R^{2}$ for the biaxial tensile tests at three different stretching strain ratios ($k=\frac{\varepsilon_{weft}}{\varepsilon_{warp}}$, *k* = 2, 1, 0.5) all increasing to 0.99. The results of this paper confirm that this new polyconvex invariant system can be used to simulate plain carbon fiber-woven fabrics.

## Figures and Tables

**Table 1:** Material A-strain energy density parameters *W*_1_ and determination coefficient *R*^2^.

Material Parameters (Mpa)	$a_{3}$	$a_{4}$	$a_{5}$	$a_{6}$	$R^{2}$
Material A	1212.833	−1434.922	949.724	−639.628	0.99

**Table 2:** Material B-strain energy density parameters *W*_1_ and determination coefficient *R*^2^.

Material Parameters (Mpa)	$a_{3}$	$a_{4}$	$a_{5}$	$a_{6}$
Material B	−10.683	1.075	0.621	1.443
k	2:1	1:1	1:2	
$R^{2}$	0.99	0.98	0.97	

**Table 3:** Material B-strain energy density parameters *W*_2_ and determination coefficient *R*^2^.

Material Parameters (kPa)	$a_{3}$	$a_{4}$	$a_{5}$	$a_{6}$	$a_{7}$	$a_{8}$
Material B	−17.9810	1.3142	−4.9073	0.7657	5.8539	9.0914
k	2:1	1:1	1:2			
$R^{2}$	0.99	0.99	0.99			

## Data Availability

Data are contained within the article.

## References

[1] Zheng G., He Z., Wang K., Liu X., Luo Q., Li Q., Sun G. (2021). On failure mechanisms in CFRP/Al adhesive joints after hygrothermal aging degradation following by mechanical tests. Thin-Walled Struct..

[2] Wang P., Hamila N., Pineau P., Boisse P. (2014). Thermomechanical analysis of thermoplastic composite prepregs using bias-extension test. J. Thermoplast. Compos. Mater..

[3] Härtel F., Harrison P. (2015). Evaluation of normalisation methods for uniaxial bias extension tests on engineering fabrics. Compos. Part A Appl. Sci. Manuf..

[4] Willems A., Lomov S.V., Verpoest I., Vandepitte D. (2007). Picture frame shear tests on woven textile composite reinforcements with controlled pretension. Esaform Conf. Mater. Forming. AIP Conf. Proc..

[5] Nosrat-Nezami F., Gereke T., Eberdt C., Cherif C. (2014). Characterisation of the shear–tension coupling of carbon-fibre fabric under controlled membrane tensions for precise simulative predictions of industrial preforming processes. Compos. Part A Appl. Sci. Manuf..

[6] Harrison P., Abdiwi F., Guo Z., Potluri P., Yu W. (2012). Characterising the shear–tension coupling and wrinkling behaviour of woven engineering fabrics. Compos. Part A Appl. Sci. Manuf..

[7] Cao J., Akkerman R., Boisse P., Chen J., Cheng H., de Graaf E., Gorczyca J., Harrison P., Hivet G., Launay J. (2008). Characterization of mechanical behavior of woven fabrics: Experimental methods and benchmark results. Compos. Part A Appl. Sci. Manuf..

[8] Tabatabaei S., Lomov S., Verpoest I. (2014). Assessment of embedded element technique in meso-FE modelling of fibre reinforced composites. Compos. Struct..

[9] Guan W., Dai Y., Li W., Qu Y., He P. (2022). An improved semi-discrete approach for simulation of 2.5D woven fabric preforming. Compos. Struct..

[10] Wineman A.S., Pipkin A.C. (1964). Material symmetry restrictions on constitutive equations. Arch. Ration. Mech. Anal..

[11] Pipkin A.C., Wineman A.S. (1963). Material symmetry restrictions on non-polynomialconstitutive equations. Arch. Ration. Mech. Anal..

[12] Takamizawa K., Hayashi K. (1987). Strain energy density function and uniform strainhypothesis for arterial mechanics. J. Biomech..

[13] Fung Y.C., Fronek K., Patitucci P. (1979). Pseudoelasticity of arteries and the choice of its mathematical expression. Am. J. Physiol.-Heart Circ. Physiol..

[14] Holzapfel G.A., Weizsäcker H.W. (1998). Biomechanical behavior of the arterial wall and its numerical characterization. Comput. Biol. Med..

[15] Guo Z.Y., Peng X.Q., Moran B. (2006). A composites-based hyperelastic constitutive model for soft tissue with application to the human annulus fibrosus. J. Mech. Phys. Solids.

[16] Aimène Y., Vidal-Sallé E., Hagège B., Sidoroff F., Boisse P. (2010). A Hyperelastic Approach for Composite Reinforcement Large Deformation Analysis. J. Compos. Mater..

[17] Islam S., Zhalmuratova D., Chung H.-J., Kim C.I. (2020). A model for hyperelastic materials reinforced with fibers resistance to extension and flexure. Int. J. Solids Struct..

[18] Yao Y., Huang X., Peng X., Gong Y. (2016). Anisotropic hyperelastic constitutive model with biaxial tension coupling for woven fabric composites. Acta Mater. Compos. Sin..

[19] Palit A., Bhudia S.K., Arvanitis T.N., Turley G.A., Williams M.A. (2018). In vivo estimation of passive biomechanical properties of human myocardium. Med. Biol. Eng. Comput..

[20] Cai R., Holweck F., Feng Z.-Q., Peyraut F. (2017). A simple polyconvex strain energy density with new invariants for modeling four-fiber family biomaterials. Int. J. Solids Struct..

[21] Ta A.-T., Labed N., Holweck F., Thionnet A., Peyraut F. (2013). A new invariant based method for building biomechanical behavior laws–application to an anisotropic hyperelastic material with two fiber families. Int. J. Solids Struct..

[22] Ball J.M. (1976). Convexity conditions and existence theorems in nonlinear elasticity. Arch. Ration. Mech. Anal..

[23] Cai R., Holweck F., Feng Z.-Q., Peyraut F. (2021). Integrity basis of polyconvex invariants for modeling hyperelastic orthotropic materials—Application to the mechanical response of passive ventricular myocardium. Int. J. Non-Linear Mech..

[24] Kakavas P.A., Giannopoulos G.I., Anifantis N.K. (2007). Mixed Finite Element Analysis of Elastomeric Butt-Joints. ASME J. Eng. Mater. Technol..

[25] Cai R., Hu L., Holweck F., Peyraut F., Feng Z.Q. (2022). Convexity, polyconvexity and finite element implementation of a four-fiber anisotropic hyperelastic strain energy density—Application to the modeling of femoral, popliteal and tibial arteries. Comput. Methods Appl. Mech. Eng..

[26] Huang X. (2016). An Anisotropic Hyperelastic Constitutive Model and Forming Simulation for Carbon Woven Fabrics. Master’s Thesis.

[27] Mooney M. (1940). A theory of large elastic deformation. J. Appl. Phys..

[28] Rivlin R.S. (1948). Large elastic deformations of isotropic materials. IV. Further developments of the general theory. Philos. Trans. R. Soc. London Ser. A Math. Phys. Eng. Sci..

